# Chitosan‐based scaffold augmentation to microfractures: Stable results at mid‐term follow‐up in patients with patellar cartilage lesions

**DOI:** 10.1002/jeo2.12065

**Published:** 2024-06-23

**Authors:** Luca De Marziani, Angelo Boffa, Luca Andriolo, Alessandro Di Martino, Giuseppe Filardo, Stefano Zaffagnini

**Affiliations:** ^1^ Clinica Ortopedica e Traumatologica 2 IRCCS Istituto Ortopedico Rizzoli Bologna Italy; ^2^ Applied and Translational Research (ATR) Center IRCCS Istituto Ortopedico Rizzoli Bologna Italy; ^3^ Department of Surgery, EOC Service of Orthopaedics and Traumatology Lugano Switzerland; ^4^ Faculty of Biomedical Sciences Università della Svizzera Italiana Lugano Switzerland

**Keywords:** cartilage, chitosan, knee, patella, scaffold

## Abstract

**Purpose:**

Patellar cartilage lesions are a frequent and challenging finding in orthopaedic clinical practice. This study aimed to evaluate a chitosan‐based scaffold's mid‐term clinical and imaging results patients with patellar cartilage lesions.

**Methods:**

Thirteen patients (nine men, four women, 31.3 ± 12.7 years old) were clinically evaluated prospectively at baseline, 12, 24 and at a final minimum follow‐up of 60 months (80.2 ± 14.7) with International Knee Documentation Committee (IKDC) subjective, Knee Injury and Osteoarthritis Outcome Score and Tegner scores. A magnetic resonance analysis was performed at the last follow‐up using the Magnetic resonance Observation of CArtilage Repair Tissue (MOCART) 2.0 score.

**Results:**

An overall significant clinical improvement in the scores was observed from baseline to all follow‐ups, with stable clinical results from 24 months to the mid‐term evaluation. The IKDC subjective score passed from 46.3 ± 20.0 at baseline to 70.1 ± 21.5 at the last follow‐up (*p* = 0.029). Symptoms' duration before surgery negatively correlated with the clinical improvement from baseline to the final follow‐up (*p* = 0.013) and sex influenced the improvement of activity level from the preoperative evaluation to the final follow‐up, with better results in men (*p* = 0.049). In line with the clinical findings, positive results were documented in terms of cartilage repair quality with a mean MOCART 2.0 score of 72.4 ± 12.5.

**Conclusions:**

Overall, the use of this chitosan‐based scaffold provided satisfactory results with a stable clinical improvement up to mid‐term follow‐up, which should be confirmed by further high‐level studies to be considered a suitable surgical option to treat patients affected by patellar cartilage lesions.

**Level of Evidence:**

Level IV, prospective case series.

**Abbreviations**
ACL: anterior cruciate ligament
BMI: body mass index
ICRS: International Cartilage Repair Society
IKDC: International Knee Documentation Committee
KOOS: Knee Injury and Osteoarthritis Outcome Score
MOCART: Magnetic resonance Observation of CArtilage Repair Tissue
MPFL: medial patello‐femoral ligament
MPTL: medial patello‐tibial ligament
MR: magnetic resonance

## INTRODUCTION

Patellar cartilage lesions are a frequent finding in orthopaedic clinical practice, with a 36% occurrence documented during knee arthroscopy [8, 51]. These lesions can lead to functional impairment, pain, inflammation and eventually the development of osteoarthritis [2, 12]. If not properly treated, this may result in invalidating conditions requiring invasive procedures like metal replacement [10]. Thus, several surgical techniques have been developed to repair the joint surface and prevent further joint damage. Among these, the microfracture technique is the first‐line technique for small lesions and it is frequently used and considered a reference for comparison of other cartilage treatments [4, 26, 29]. During microfracture, penetration of the subchondral bone plate into the cartilage defect leads to bleeding and the subsequent formation of a fibrin clot, which fills the defect covering the exposed bone surface [48]. However, although some studies have shown that microfractures can achieve good clinical results in the short term [45], long‐term clinical results appear to be suboptimal [31, 34].

Research efforts have been invested to improve the microfracture approach, aiming at overcoming its limits by increasing the stability of the fibrin clot and possibly enhancing tissue quality and durability of the functional outcome [6]. In this light, several scaffolds have been developed based on different molecules, such as chitosan [18, 42, 49]. Chitosan, a deacetylated chitin derivative found in the exoskeletons of crustaceans and insects [36], is biocompatible, biodegradable and a suitable scaffold for the proliferation and differentiation of chondrocytes [9, 35]. An injectable chitosan‐based scaffold has been used as an augmentation of the microfracture technique to stabilise fibrin clots, provide a structural framework for subsequent cellular ingrowth and impede clot retraction. This scaffold has already been tested in clinical practice, with satisfactory results for the treatment of cartilage lesions localised to the femoral condyles [43, 46, 47]. On the other hand, only a few data are available on the clinical and imaging results of this scaffold for the treatment of challenging patellar cartilage lesions; while promising results have been shown at short‐term follow‐up [5, 38], there is still a lack of evidence at mid‐term follow‐up to confirm the durability of the clinical benefit.

This study aimed to evaluate the mid‐term clinical and imaging results of this chitosan‐based scaffold augmented to microfractures in patients with patellar cartilage lesions.

## MATERIAL AND METHOD

This study presents the clinical and imaging assessment at the mid‐term follow‐up of a series of patients affected by patellar cartilage lesions previously evaluated for up to 24 months [38]. This study was approved by the hospital Ethics Committee of the IRCCS Istituto Ortopedico Rizzoli, Bologna, Italy (protocol number 571/2023/Oss/IOR) and informed consent of all patients was obtained. Inclusion criteria were: Patients with patellar chondral defects classified as grades 3–4 International Cartilage Repair Society and presenting knee clinical symptoms, such as pain, swelling or giving way. Exclusion criteria were represented by shellfish and chitosan allergy, patellofemoral chondral kissing lesions, untreated tibiofemoral malalignment (>5° on full‐length standing anteroposterior radiographs), rheumatic and autoimmune diseases, coagulopathies or arthropathies secondary to genetic or metabolic diseases.

A total of 15 patients were enroled and treated from 2015 and 2018 in a research clinic focused on knee cartilage restoration. Among these, two patients were lost to follow‐up at 2 and 24 months after the treatment, while 13 patients were clinically and radiologically evaluated prospectively for a minimum follow‐up of 60 months. Among these, nine patients were men and four women, with a mean age at the time of surgery of 31.3 ± 12.7 years. Five patients had previous surgical procedures, in detail: two anterior cruciate ligament (ACL) reconstructions, including one case of subsequent ACL revision, two meniscectomies, one microfracture for femoral lesion, one lateral release, one chondro‐abrasion of patellar cartilage and one case of patellar fracture treated with tension band with Kirschner wire. Eight patients had combined surgery, in detail: one ACL reconstruction, three medial patello‐femoral ligament reconstructions, one medial patello‐tibial ligament reconstruction, two tibial tubercle transfer and in one case a combined medial tibial plateau chondral defect was treated with the same scaffold. Further baseline characteristics of patients and patellar cartilage defects are listed in Table 1.

**Table 1:** Included patients' characteristics.

Sex (M/W)	9/4
Age (years)	31.3 ± 12.7
BMI (kg/m^2^)	24.6 ± 3.8
Lesion size (cm^2^)	3.0 ± 0.8
Symptoms duration (months)	24.6 ± 35.6
Symptoms onset	Acute: 5‐Chronic: 8
Previous knee surgery (yes/no)	5/8
Associated knee surgery (yes/no)	8/5

*Note*: Values are expressed as mean ± standard deviation.

Abbreviations: BMI, body mass index; M, men; W, women.

### Procedure and patient's evaluations

The surgical technique involved a one‐stage arthrotomy combining the chitosan‐based scaffold (CARGEL Bioscaffold, formerly BST‐CarGel; Smith & Nephew) with microfractures, as previously reported [38]. In brief, the patellar cartilage defect was exposed through a medial para‐patellar approach and then prepared to perform the microfracture procedure, according to Steadman et al. [44]. The liquid bio‐scaffold was prepared following the manufacturer's instructions, applied to fill the defect and allowed to polymerise in the lesion for 15 min to stabilise the clot before closure. Postoperative rehabilitation included 24‐h leg immobilisation, early passive mobilisation after 2 weeks and gradual progression in rehabilitation exercises, allowing high‐impact activities at 12 months postsurgery.

Patients were clinically evaluated before the cartilage procedure and at 12, 24 and a minimum follow‐up of 60 months (mean of 80.2 ± 14.7 months). Patients were assessed through the International Knee Documentation Committee (IKDC) subjective score (0–100), the Knee Injury and Osteoarthritis Outcome Score (KOOS) (0–100) and the Tegner activity scale (1–10). Patient judgement of the treatment was also recorded at the last follow‐up using a specific question: ‘Compared to the baseline status, how would you rate the treated knee now?’. The responses were recorded using a six‐point scale: ‘full recovery’, ‘much better’, ‘somewhat better’, ‘about the same’, ‘somewhat worse’ and ‘much worse’. Treated knees were also evaluated through magnetic resonance (MR) imaging at the last follow‐up to assess the quality of the regenerated cartilage tissue using the Magnetic resonance Observation of CArtilage Repair Tissue (MOCART) 2.0 score [40]. The procedure was considered a failure if the patient required further surgical treatment of the patellar cartilage due to symptoms caused by the same lesion. For failed patients, the worst clinical evaluation between baseline and the last available follow‐up was considered for the following assessments.

### Statistical analysis

All continuous data were expressed in terms of the mean and the standard deviation of the mean; the categorical data were expressed as frequency and percentages. The analysis of variance repeated measures were performed to assess the score's differences along the follow‐up times; this test was followed by a post‐hoc pairwise comparison with the Sidak test for multiple comparisons to check the differences between the follow‐up times. The Mann−Whitney nonparametric test, evaluated by the Monte Carlo method for small samples was performed to assess the between groups differences in the scores. The Spearman correlation was used to assess the influence of age, body mass index (BMI) and symptom duration on the scores. For all tests, *p* < 0.05 was considered significant.

All statistical analysis was performed using SPSS v.19.0 (IBM Corp.).

## RESULTS

**Figure 1:**
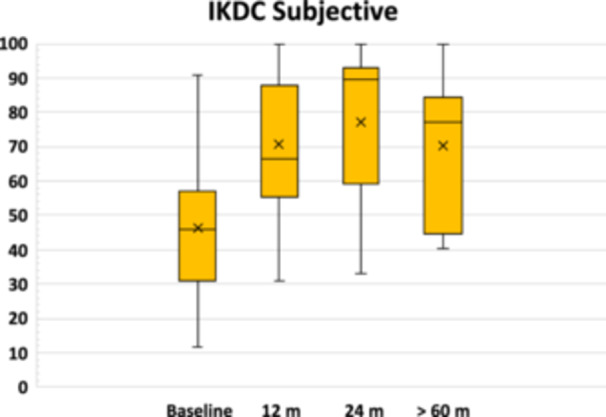
The International Knee Documentation Committee (IKDC) subjective score. The box and whisker plots show the median, mean and quartiles.

**Figure 2:**
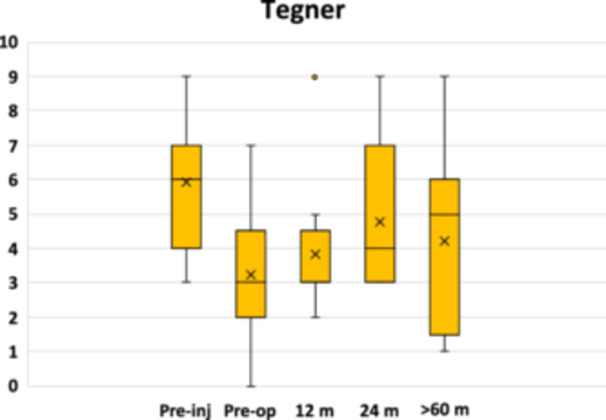
Tegner activity scale trend after treatment. The box and whisker plots show the median, mean and quartiles.

An overall significant clinical improvement in the scores was observed from baseline to all follow‐ups, with stable clinical results from 24 months to the mid‐term evaluation. In particular, the IKDC subjective score (Figure 1) remained stable from 77.1 ± 21.1 at 24 months to 70.1 ± 21.5 at the last follow‐up (n.s.), with significantly higher values compared to the baseline value of 46.3 ± 20.0 (*p* = 0.029). Similar trends were reported for all the KOOS subscales, as reported in Table 2. The Tegner activity scale (Figure 2) did not statistically improve from the preoperative value of 3.2 ± 1.9 to all follow‐ups. However, while the preoperative level was significantly lower than the preinjury level (*p* = 0.009), there were no longer statistically significant differences with the preinjury score (5.9 ± 1.8, n.s.) at 24 months (4.8 ± 2.2) and the last follow‐up (4.2 ± 2.3). Regarding treatment judgement at the last follow‐up, four patients reported ‘full recovery’, seven patients considered ‘improved’ their treated knee (five ‘much better’ and two ‘somewhat better’), while two patients reported ‘about the same’.

**Table 2:** Clinical score values at basal level and 12, 24 and ≥60 months of follow‐up.

Score	Baseline	12 months	24 months	≥60 months	ANOVA test
IKDC subjective	46.3 ± 20.0	70.8 ± 20.6	77.1 ± 21.1	70.1 ± 21.5	0.017
KOOS pain	66.8 ± 22.2	87.5 ± 11.9	89.9 ± 13.5	88.2 ± 11.1	0.014
KOOS symptoms	58.0 ± 22.4	81.0 ± 14.3	81.3 ± 16.5	80.5 ± 12.8	0.049
KOOS ADL	75.0 ± 20.3	88.3 ± 16.1	93.7 ± 11.0	92.9 ± 7.8	0.039
KOOS sport/rec	27.7 ± 27.7	71.1 ± 24.2	71.2 ± 33.6	62.3 ± 24.2	0.003
KOOS QoL	24.9 ± 20.0	67.7 ± 28.3	71.7 ± 23.0	65.6 ± 28.7	0.002
Tegner activity scale	3.2 ± 1.9	3.9 ± 1.8	4.8 ± 2.2	4.2 ± 2.5	n.s.

*Note*: Values are expressed as mean and standard deviation.

Abbreviations: ADL, activities of daily living; ANOVA, analysis of variance; IKDC, International Knee Documentation Committee; KOOS, Knee injury and Osteoarthritis Outcome Score; n.s., not significant; QoL, quality of life; Sport/Rec, sport and recreation.

One patient was considered a surgical failure, being treated with subchondroplasty at 79 months of follow‐up. Moreover, one patient received an intra‐articular injection of adipose tissue‐derived mesenchymal stromal cells in the treated knee at 74 months of follow‐up, while another patient was conservatively treated for a meniscal lesion reported during physical activity at 50 months of follow‐up and without persistence of symptoms at the last clinical evaluation.

Further analysis was performed to determine the parameters influencing the clinical improvement from baseline to the final follow‐up. Symptoms' duration before surgery negatively correlated with the clinical improvement from baseline to the final follow‐up in terms of IKDC subjective score (*p* = 0.013, *⍴* = −0.665), KOOS Symptoms (p = 0.036, *⍴* = − 0.584) and Tegner activity scale (*p* = 0.030, *⍴* = − 0.600), with higher improvements in patients with lower symptoms' duration. Sex influenced the improvement of activity level from the preoperative evaluation to the final follow‐up, with higher improvements in men compared to women (+1.9 ± 2.9 vs. −1.0 ± 1.4, *p* = 0.049). Similarly, men reported better results in terms of differences between the preinjury level and the final follow‐up (−0.7 ± 2.2 vs. −4.0 ± 1.8, *p* = 0.035). Age, BMI, lesion size, aetiology, previous surgery and associated surgery did not influence the clinical improvement from baseline to the last follow‐up in this series.

**Figure 3:**
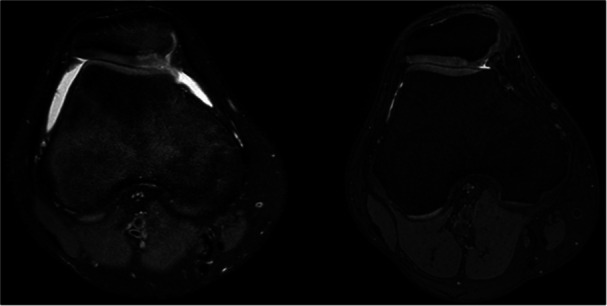
On the left, an axial pre‐perative magnetic resonance imaging shows a patellar cartilage lesion. On the right, the 5‐year postoperative magnetic resonace imaging after chitosan‐based scaffold implantation showed an improvement of most Magnetic resonance Observation of CArtilage Repair Tissue 2.0 variables evaluated.

As described in Table 3, the cartilage repair processes evaluated with MR showed a total mean MOCART 2.0 score of 72.4 ± 12.5 at the final follow‐up (Figure 3).

**Table 3:** Magnetic resonance Observation of CArtilage Repair Tissue 2.0 score at minimum 60 months of follow‐up.

**Variables**	**% of lesions**
Volume fill of cartilage defect	
Complete filling OR minor hypertrophy (100%–150%)	43
Major hypertrophy (≥150%) OR 75%–99% filling	57
50%–74% filling	/
25%–49% filling	/
<25% filling OR complete delamination in situ	/
Integration into adjacent cartilage	
Complete	28
Split‐like defect ≤ 2 mm	72
Defect >2 mm but <50% of repair tissue length	/
Defect ≥50% of repair tissue length	/
Surface of the repair tissue	
Intact	14
Damaged: <50% of the repair tissue diameter	86
Damaged: ≥50% of the repair tissue diameter	/
Structure of the repair tissue	
Homogeneous	43
Inhomogeneous	57
Signal intensity of the repair tissue	
Normal	14
Minor abnormal: minor hyperintense OR minor hypointense	86
Severely abnormal	/
Bony defect or bony overgrowth	
No bony defect or bony overgrowth	72
Bony defect: depth <thickness of adjacent cartilage OR overgrowth <50% of adjacent cartilage	28
Bony defect: depth ≥thickness of adjacent cartilage OR overgrowth ≥50% of adjacent cartilage	/
Subchondral changes	
No major subchondral changes	44
Minor oedema‐like marrow signal: maximum diameter <50% of the repair tissue diameter	28
Severe oedema‐like marrow signal: maximum diameter ≥50% of the repair tissue diameter	14
Subchondral cysts ≥5 mm OR osteonecrosis‐like signal	14

## DISCUSSION

The main finding of this study is that this chitosan‐based scaffold provided stable clinical results up to mid‐term follow‐up in patients affected by patellar cartilage lesions.

The treatment of patellar cartilage lesions represents a challenge for orthopaedic surgeons with poorer outcomes and increased failure rates when compared with the treatment of chondral defects of the femoral condyles and trochlea [14, 22, 23, 24]. Currently, different surgical strategies have been proposed to address cartilage lesions of the patella, as reported by a recent systematic review of clinical studies focused on the treatment of isolated chondral patellar defects [18]. The most widely studied treatments for cartilage lesions of the patella are osteochondral autologous transplantation and autologous chondrocyte transplantation [18]. However, no treatments demonstrated fully satisfactory results when addressing patellar cartilage lesions, prompting research efforts to seek new more effective alternatives, including cell‐free cartilage scaffold implantation procedures [31, 39].

Scaffolds can be based on different types of matrices, such as collagen [3], hyaluronic acid [50] or chitosan, as the scaffold evaluated in the current study [38]. This chitosan‐based scaffold already showed to provide promising clinical results and improved cartilage repair compared to microfractures for the treatment of condylar cartilage lesions [41, 43, 47]. On the other side, only a few data are available on the use of this scaffold for the more challenging patellar cartilage lesions [43, 46]. Among these, Calvo et al. [5] conducted a retrospective observational study on cartilage lesions of the patello‐femoral joint evaluating 15 patients treated with this bioscaffold combined with microfractures. These authors reported positive clinical and imaging results at a mean final follow‐up of 33 months, although only four patients were affected by isolated patellar cartilage lesions, which makes it impossible to draw clear conclusions on the effectiveness of this treatment in this site.

This study focused on 15 patients, all with cartilage lesions of the patella treated with this chitosan scaffold, and evaluated prospectively up to a minimum follow‐up of 60 months. This bioscaffold provided stable results over time, with no significant worsening from 2 years to the last follow‐up. Similarly, MR also showed positive results in terms of tissue repair evaluated through the MOCART 2.0 score. This is an important finding that testifies the persistence of the repair tissue quality at mid‐term follow‐up, which was also compared to values previously documented in the short‐term [38]. Still, previous studies showed a poor correlation between this score and the patient's symptoms [13, 27]. Thus, while MR remains an essential diagnostic tool for the evaluation of cartilage lesions, patients' function and symptoms remain the primary outcome when evaluating the potential of a cartilage procedure [28].

The current study also showed that patients with less symptom duration before surgery had better clinical outcomes and higher clinical improvement following the implantation of the chitosan‐based scaffold. The time between the onset of symptoms and its treatment is a fundamental parameter, especially for patella lesions. In a previous study by Pettit et al., it has been demonstrated how untreated cartilaginous lesions increase by 0.11 cm^2^ every month and how cartilaginous lesions of the patella tend to progress more quickly towards high‐grade lesions [37]. The worse clinical results found in patients with pain for longer could be linked not only to an increase in the size of the original cartilage defect but also to degenerative processes of the surrounding cartilage [11, 32, 37]. There is a complex interaction of macroscopic and microscopic factors, such as altered edge stress on the defect that can lead to increased pressure, expansion and consequent increased loading on the subchondral bone [21]. The subchondral bone plays a fundamental role, especially for those treatments based on the use of microfractures and in this sense lesions involving subchondral bone can lead to lower regenerative capacities [52]. Furthermore, this degenerative process can trigger joint inflammation inducing chondrocytes to produce degradative enzymes of the extracellular matrix and inhibit both tissue repair and regeneration [1]. In this perspective, cartilage lesions of the patella should be treated more quickly than others due to their greater potential for progression.

The responses to the chitosan‐based scaffold procedure changed also in relation to gender. Men presented a greater increase in the activity level after treatment and reached better results in terms of sport activity at the last follow‐up compared to women. The negative effect of the female gender on the results of cartilage regeneration procedures has been already reported in previous studies focused on cartilage treatments [16, 17]. Women often have a different chondral injury pattern with more frequently unfavourable conditions related to the cause, site and comorbidities of the injury. Moreover, the biomechanics of the patello‐femoral joint may differ between men and women, with greater variations in contact pressures during knee flexion highlighted in women compared to men, suggesting sexual differences in contact areas and patello‐femoral pressures [7, 25]. Furthermore, women often start from lower scores of knee function and activity level compared to men [15, 30]. Men and women have numerous biomechanical and biological differences, and analysing the peculiarities of men and women is a pressing aspect of orthopaedics [11]. The importance of studying gender‐based specific characteristics and outcomes is confirmed by the lower scores documented in the current study in women treated with this bioscaffold for patellar defects. However, future studies with higher sample sizes should confirm the influence of gender on clinical outcomes provided by this bioscaffold for patellar defects.

This study has some limitations. First, the study design and the lack of a control group do not allow us to draw clear conclusions on the added benefits offered by this chitosan‐based scaffold compared to the microfracture technique alone. However, this is one of the few studies currently available in the literature evaluating the clinical improvement of this scaffold in a challenging site such as the patella, whereas the potential of this approach compared to the microfracture technique was previously tested in other knee sites. Moreover, the very small patient cohort, although adequate to analyse the primary outcome, could impair statistical power to detect further significant differences and the identification of correlations between patient and lesion characteristics such as sex, time to surgery, age, BMI, lesion size, aetiology, previous surgery and associated surgery and clinical outcomes. Additionally, it is important to emphasise the heterogeneity among the evaluated patients, particularly, in terms of previous and concurrent surgeries, which could be an additional confounding factor. On the other hand, this cohort represents the real‐world scenario and thus could provide results closer to those that could be expected in clinical practice. Finally, the follow‐up of the study provides solid data on the treatment's potential for patient improvement in the medium term, and further information is required to assess the effect of this scaffold at longer follow‐up.

Based on the results of this study in terms of clinical and imaging outcomes up to mid‐term follow‐up, this one‐stage cell‐free chondral procedure can be considered a viable option for the treatment of patellar defects. This type of treatment demonstrated promising mid‐term results both from an imaging and clinical point of view. Despite some adverse events previously documented in the short term [38], it proved to be a treatment with a low failure rate similar to more expensive treatments for cartilage lesions of the patella [19, 20, 33]. Nevertheless, higher‐level studies are needed to confirm the results of this pilot study and compare them with those of other available treatments.

## CONCLUSIONS

The use of this chitosan‐based scaffold augmented with microfractures provided a stable clinical improvement up to mid‐term follow‐up in patients affected by patellar cartilage lesions. Moreover, the MR evaluation is in line with the clinical findings, showing positive results in terms of cartilage repair quality. Higher‐level studies should further investigate and confirm these findings and the potential of this scaffold for the treatment of patellar cartilage lesions.

## AUTHOR CONTRIBUTIONS

**Luca De Marziani**: Methodology; data curation; writing—original draft preparation. **Angelo Boffa**: Methodology; data curation; writing—original draft preparation. **Luca Andriolo**: Methodology; writing—review and editing. **Alessandro Di Martino**: Methodology, writing—review and editing. **Giuseppe Filardo**: Conceptualisation; writing—review and editing; supervision. **Stefano Zaffagnini**: Supervision. All authors have read and agreed to the published version of the manuscript.

## CONFLICT OF INTEREST STATEMENT

Stefano Zaffagnini reports nonfinancial support from personal fees from I + SRL and grants from Fidia Farmaceutici SPA, Cartiheal Ltd., IGEA clinical biophysics, BIOMET and Kensey Nash, outside the submitted work. The funders had no role in the design of the study; in the collection, analyses or interpretation of the data; in the writing of the manuscript or in the decision to publish the results. The remaining authors declare no conflict of interest.

## ETHICS STATEMENT

The study was approved by the hospital Ethics Committee of the IRCCS Istituto Ortopedico Rizzoli, Bologna, Italy (protocol number 571/2023/Oss/IOR). Informed consent of all patients was obtained.
